# Conformal SiO_2_ coating of sub-100 nm diameter channels of polycarbonate etched ion-track channels by atomic layer deposition

**DOI:** 10.3762/bjnano.6.48

**Published:** 2015-02-16

**Authors:** Nicolas Sobel, Christian Hess, Manuela Lukas, Anne Spende, Bernd Stühn, M E Toimil-Molares, Christina Trautmann

**Affiliations:** 1 Eduard-Zintl-Institut für Anorganische und Physikalische Chemie, Technische Universität Darmstadt, Alarich-Weiss-Str. 8, 64287 Darmstadt, Germany; 2 Institute of Condensed Matter Physics, Technische Universität Darmstadt, Hochschulstraße 8, 64289 Darmstadt, Germany; 3 Materials Research Department, GSI Helmholtz Centre for Heavy Ion Research, Planckstr. 1, 64291 Darmstadt, Germany; 4 Material- und Geowissenschaften, Technische Universität Darmstadt, Alarich-Weiss-Str. 2, 64287 Darmstadt, Germany

**Keywords:** atomic layer deposition (ALD), ion-track technology, nanochannels, polycarbonate, silica (SiO_2_), small angle X-ray scattering (SAXS), track-etched channels, X-ray photoelectron spectroscopy (XPS)

## Abstract

Polycarbonate etched ion-track membranes with about 30 µm long and 50 nm wide cylindrical channels were conformally coated with SiO_2_ by atomic layer deposition (ALD). The process was performed at 50 °C to avoid thermal damage to the polymer membrane. Analysis of the coated membranes by small angle X-ray scattering (SAXS) reveals a homogeneous, conformal layer of SiO_2_ in the channels at a deposition rate of 1.7–1.8 Å per ALD cycle. Characterization by infrared and X-ray photoelectron spectroscopy (XPS) confirms the stoichiometric composition of the SiO_2_ films. Detailed XPS analysis reveals that the mechanism of SiO_2_ formation is based on subsurface crystal growth. By dissolving the polymer, the silica nanotubes are released from the ion-track membrane. The thickness of the tube wall is well controlled by the ALD process. Because the track-etched channels exhibited diameters in the range of nanometres and lengths in the range of micrometres, cylindrical tubes with an aspect ratio as large as 3000 have been produced.

## Introduction

Track-etched membranes are fabricated by heavy-ion irradiation of polymer foils and subsequent chemical etching of the ion tracks [1–2]. By controlling the etching parameters, cylindrical and conical channels can be fabricated by symmetric and asymmetric etching, respectively [3]. The diameter of the channels increases with the etching time and can be adjusted in a controlled manner between ca. 20 nm and several micormetres. Exposure of the irradiated foils to UV light prior to etching results in track-etched membranes with a very narrow size distribution [4–6]. Typically, polymers such as polycarbonate (PC), polyethylene terephthalate, and polyimide are employed as templates. Hydrophilicity is sometimes improved by immersing the etched membrane in a surfactant solution such as polyvinylpyrrolidone. The addition of surfactants, however, is known to affect the final geometry of the channels, which changes, for example, from a cylindrical to a cigar-like shape [5]. Etched ion-track membranes are widely used for a broad range of scientific and industrial applications. Recent research activities with single channel as well as with multichannel membranes focus on the chemical modification of channel walls to investigate ionic and molecular transport properties [7–8], with the aim to develop sensors based on nanochannels with tailored surface [9]. Track-etched membranes are also most suitable for the synthesis of nanowires or nanotubes by electrochemical [10–11] or electroless deposition [12–13].

Transport processes in narrow nanochannels are strongly affected by the surface characteristics of these channels. In the case of etched polycarbonate, carboxylate groups (–COO^−^) are present at the surface [14]. Specific channel properties such as diameter and conformation variations due to dangling bonds, swelling, or surface charge variations from pH changes of the solution, are to a large extent unknown but can influence ion transport and the control of surface modification steps in a crucial manner. A homogeneous conformal coating of the entire membrane by an inorganic material would provide a well-defined reference surface. Coating by means of atomic layer deposition (ALD) is a novel approach to reduce the diameter of track-etched nanochannels in a controlled manner. Channels with diameter below 10 nm are extremely interesting to study water and ion transport in confinement [15–16]. Coated templates are also attractive to synthesize extremely thin nanowires for the investigation of finite size and quantum size effects [17].

Atomic layer deposition is based on cycles of self-limiting chemical reactions between the gas-phase precursor molecules and a solid surface, providing very thin uniform films on arbitrarily shaped materials. The number of cycles determines the thickness of the deposited layer. Tailored coating with various materials such as metals (e.g., Pt) or oxides (e.g., ZnO, Al_2_O_3_, TiO_2_) can be used to adjust specific surface properties such as hydrophilicity or catalytic activity. Conform ALD coatings have been reported for various porous materials including powders [18] and porous membranes such as anodic aluminum oxide (AAO) [19–24] and track-etched membranes [25–28]. For example, channels with diameters of about 80 nm and lengths of about 700 nm of AAO membranes were successfully coated with a 20 nm thick SiO_2_ layer through the consecutive pulsed appliciation of 3-aminopropyltriethoxysilane, water and ozone [22]. Velleman et al. applied the precursor couple tri(*tert*-butoxy)silanol and trimethylaluminium to coat 20, 100, and 200 nm diameter AAO channels with silica [23]. Channels (diameter 70 nm, length 2 µm) of polyimide templates fabricated by AAO-assisted dry etching were coated with Al_2_O_3_ [24]. Commercial track-etched membranes were successfully ALD-coated with TiO_2_, ZrO_2_, and Al_2_O_3_ [25–28].

In this contribution, we present the successful SiO_2_ coating of high aspect ratio nanochannels in PC track-etched membranes. After ALD coating, the surfaces are covered with silanol (Si–OH) groups that are also characteristic for other porous systems such as silica nanopores sculptured by electron or kiloelectronvolt-ion beams, mesoscopic silica, or silica nanotubes. Such systems are of interest for numerous applications due to their facile surface functionalization, hydrophilic nature, and biocompatibility [29]. For the deposition of SiO_2_ on polymer surfaces, one must guarantee the thermal stability of the polymer. We thus developed a low-temperature process by using silicon tetrachloride (SiCl_4_) and water as precursors and pyridine as catalyst. For PC track-etched membranes this approach successfully produces uniform and conformal SiO_2_ coatings. To evidence the conformity of the developed ALD process, coated membranes were analyzed by small angle X-ray scattering (SAXS). Pronounced oscillations of the X-ray intensity as a function of the scattering vector demonstrate both an excellent conformity and uniformity of the SiO_2_ layers in the channels, and a narrow size distribution of the cylindrical channels in the template. This result is further confirmed by high resolution scanning electron microscopy (HRSEM) of nanotubes released from the PC matrix by dissolving the polymer. The chemical composition of the deposited layer is analyzed by X-ray photoelectron spectroscopy (XPS) and Fourier-transform infrared spectroscopy (FTIR). The hydrophilicity of SiO_2_-coated PC membranes was tested by contact angle measurements.

## Experimental

**Figure 1:**
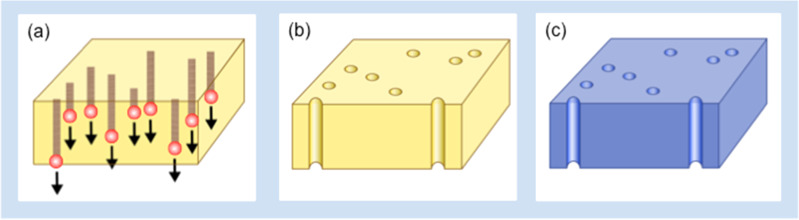
Schematics of the fabrication of SiO_2_ coated membranes: (a) irradiation of PC foil with GeV heavy ions, (b) chemical etching of ion tracks to form cylindrical nanochannels, (c) ALD conformal coating of porous membrane.

Figure 1 displays schematically the three main steps involved in the fabrication of SiO_2_ coated track-etched membranes. These include (a) irradiation of polymer foils with a well-defined number of approx. 2 GeV heavy ions, (b) chemical etching that converts each individual ion track into a cylindrical channel; the etching time determines the channel diameter (120 s etching resulted in channels of about 50 nm in diameter), and (c) SiO_2_ coating by ALD.

### Fabrication of polycarbonate track-etched membranes

Foils of 30 μm thick polycarbonate (Makrofol N, Bayer) were irradiated at the UNILAC linear accelerator of the GSI Helmholtz Centre for Heavy Ion Research (Darmstadt, Germany) using Au ions of 2 GeV kinetic energy. The exposure was performed under normal beam incidence applying a fluence of 10^9^ ions/cm^2^, which is sufficiently low to obtain non-overlapping pores. Prior to chemical etching, both sides of the foils were treated with UV light of wavelengths of 280–400 nm. This procedure is known to sensitize the ion tracks and increase the selectivity of the etching process. Etching was performed in 6 mol·L^−1^ NaOH at 50 °C followed by thorough rinsing in deionized water (Millipore Direct-QTMS). All samples were etched for 120 s resulting in cylindrical nanochannels of about 50 nm in diameter corresponding to a length-to-diameter ratio of approximately 600.

### Deposition of SiO_2_ by atomic layer deposition (ALD)

Due to the limited stability of polycarbonate foils at temperatures above approx. 413 K, we slightly modified the ALD deposition of SiO_2_ reported earlier for 700 K [30]. Employing pyridine as catalyst allowed us to lower the deposition temperature close to room temperature [31]. The coating was performed in a custom-built facility operated at 325 K. The two precursors, SiCl_4_ (99%, Sigma-Aldrich) and distilled H_2_O, were mixed with pyridine (99.8%, anhydrous, Sigma-Aldrich) as catalyst. Purified nitrogen (99.999%) at a flow rate of 200 sccm was used as purge gas. The ALD cycles consisted of a 70 s N_2_ purge, exposure to pyridine (5 s), exposure to SiCl_4_ (60 s), followed by a second 70 s N_2_ purge, exposure to pyridine (5 s), and exposure to H_2_O (60 s). Long exposure and purge times were chosen to ensure complete coating of the high-aspect-ratio nanochannels. The absolute exposures of pyridine, SiCl_4_, and H_2_O are 2.04, 7.93, and 429 mbar·s, respectively. The reaction mechanism for silica deposition with SiCl_4_ and water can be divided into the following reactions (Equation 1 through Equation 2):

[1]
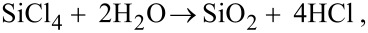


[3]
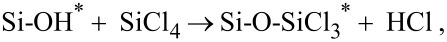


[2]
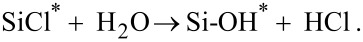


The asterisks mark surface species. Hydrogen chloride is a byproduct. It has been reported that reaction of pyridine and hydrogen chloride may lead to the formation of pyridinium salt [31].

### FTIR spectroscopy

Infrared spectra were recorded at room temperature in diffuse reflectance on a Bruker Vertex 70 system equipped with a Praying Mantis High Temperature Reaction Chamber (Harrick Scientific Prodructs, INC.). Prior to analysis, samples were heated at 493 K for 1 h in N_2_ atmosphere to remove physisorbed water. All spectra are normalized to the band at 1019 cm^−1^.

### X-ray photoelectron spectroscopy (XPS)

The SiO_2_-coated surface was analyzed by XPS using a modified LHS/SPECS EA2000 MCD system [32]. The samples were aligned normal to the analyzer and measured at pressures between 5.25 × 10^−7^ and 3.75 × 10^−8^ mbar. To prevent charging of the membrane and to ensure a constant chemical background for samples smaller than the spot size of the analyzer, the ALD-treated foils were deposited on a gold-coated quartz crystal microbalance (QCM). To account for charging effects all spectra were shifted to the Si 2p emission of SiO_2_ at 103.3 eV [33]. Spectra were corrected for the Au background before C 1s and O 1s analysis. The applied relative sensitivity factors (R.S.F) are given in Table 1 (see below) [34]. The least-square fitting analysis was performed by using a Voigt function (45% Gauss fraction).

### Small angle X-ray scattering (SAXS)

The characterisation of the pores was performed by means of small angle X-ray scattering which is a non-destructive method that yields information on the geometry and size distribution of all scattering objects (in our case about 10^6^ channels) in a given sample. The experiments were performed on a laboratory instrument consisting of a sealed X-ray tube (Panalytical) with an X-ray mirror providing Cu Kα radiation of λ = 1.54 Å. Collimated by three pinholes, the beam hits the sample mounted on a goniometer-like device, which allowed us to rotate the sample around the two axes perpendicular to the incoming beam (accuracy better than 0.01°). The scattering pattern was recorded by a two-dimensional detector (Molecular Metrology) at 150 cm distance from the sample providing an accessible range of *q*-vectors between 0.008 Å^−1^ up to 0.25 Å^−1^. Precise orientation of the sample with respect to the scattering plane is important in order to be able to interpret the data quantitatively. During SAXS analysis, the surface normal of the membrane is first orientated parallel to the incoming beam (γ = 0°). In this configuration, the beam is collinear to the longitudinal axis of the highly parallel-oriented pores and an isotropic scattering image is obtained. When the channel axis is slightly tilted with respect to the X-ray beam (e.g., γ = 20°), the SAXS patterns become highly anisotropic consisting of a narrow streak parallel to the axis of rotation. This pronounced dependence on the angle originates from the large length-to-diameter ratio of the channels. The scattered intensity, *I*(*q*), in the streak is analysed as a function of the scattering vector *q*. Information on the channel diameter is provided by fitting the *I*(*q*) data with a two-dimensional simulation code that describes the channels as cylindrical scattering objects. In the analysis parameters such as cylinder size and length dispersion, diffractometer resolution, and background subtractions are considered [35–36].

### Scanning electron microscopy (SEM)

To visualize the material deposited inside the nanochannels, the polycarbonate templates were dissolved in dichloromethane (>99.5%, Carl Roth GmbH). The released nanotubes were transferred onto standard Cu-lacey transmission electron microscopy grids. Thickness and homogeneity of the resulting nanotubes were characterized by using a high resolution scanning electron microscope (JEOL JSM-7401F) fitted with a transmitted electron detector (STEM-in-SEM).

### Contact angle measurements

Contact angles were measured on a OCA35 apparatus from Dataphysics Instruments GmbH. Distilled water with a volume of 2 µL was dropped onto the membrane surface at 21 °C and 50% relative humidity of air. Besides ALD coating, the samples were not treated otherwise prior to measurements.

## Results and Discussion

### Composition and growth mechanism

**Figure 2:**
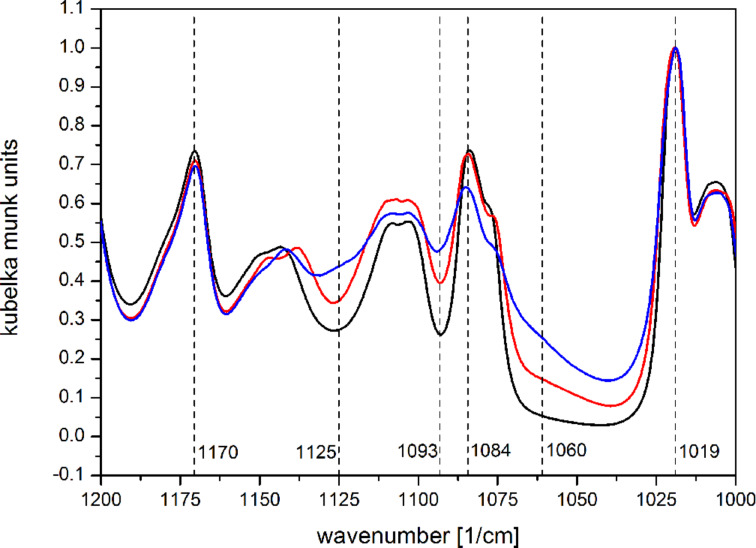
Diffuse reflectance FTIR spectra of uncoated (black) and coated (28 cycles (red), 112 cycles (blue)) polycarbonate membranes. Spectra were normalized to the band at 1019 cm^−1^.

The composition of the ALD layer is decisive for the functionality of the modified membrane, and thus was analyzed by XP and FTIR spectroscopy. Figure 2 shows diffuse reflectance FTIR spectra of a pristine PC membrane (black), and of two membranes coated with 28 (red) and 112 (blue) ALD cycles. In the wavenumber range 1000–1200 cm^−1^, the spectra are characterized by six bands at 1019, 1060, 1084, 1093, 1125, and 1170 cm^−1^. The bands at 1019, 1084, and 1170 cm^−1^ originate from polycarbonate and are attributed to the para-substituted phenyl group (1019, 1084 cm^−1^) and C–O–C stretching of the ester group (1170 cm^−1^) [37]. The intensity of bands characteristic for the asymmetric Si–O–Si stretch vibration (1060, 1093, and 1125 cm^−1^) significantly increases. The 1060 cm^−1^ band exhibits in addition a pronounced shoulder at lower wavenumbers. These bands are assigned to stretching vibrations of Si–O–Si bonds with different angles in the silica network structure [38]. The observation of these bands provides clear evidence of silica on the polycarbonate membranes. Hydroxy-related signals were not observed due to the low surface area of the samples.

The surface composition of pristine and ALD-coated samples was analyzed by XPS (Table 1). The spectra of the coated samples are characterized by major peaks at 532.7, 284.5, and 103.3 eV originating from O 1s, C 1s, and Si 2p photoemission, respectively. The strong decrease of the carbon contribution and the nearly constant O 1s/Si 2p ratio indicate the coating of the polycarbonate surface by silica for all film thicknesses.

**Table 1:** Surface composition (in atom %) of a pristine PC membranes and SiO_2_-coated membrane after different numbers of ALD cycles.

ALD	C 1s	N 1s	Cl 2p	Si 2p	O 1s
R.S.F.	0.25	0.42	0.73	0.27	0.66

0	0.83	—	—	—	0.17
28	0.22	—	—	0.22	0.56
56	0.12	—	—	0.28	0.60
84	0.19	—	—	0.24	0.57
112	0.19	0.01	0.01	0.25	0.54

Despite the use of pyridine as catalyst, impurities were only detectable for the SiO_2_ film obtained by application of 112 ALD cycles. In this case, minor peaks at 401.7 eV (N 1s) and 199.9 eV (Cl 2p) were observed, which may result from incorporated pyridinium chloride.

Detailed analysis of the Si 2p peak at 103.3 eV reveals that silicon is exclusively present as Si^4+^ (see Table 2). This conclusion is based on a comparison with literature values for Si^3+^, Si^2+^, and Si^1+^ binding energies of 102.3, 101.3, and 100.5 eV, respectively (referenced to 103.3 eV) [39]. Table 2 also shows that the ratios of the O 1s/Si 2p signal range from 2.62 to 2.12. For coatings with more than 28 ALD cycles, the ratio is rather stable at a value slightly above 2 consistent with SiO_2_. The observed peak widths (FWHM = full width at half maximum) of the O 1s and Si 2p emissions agree with literature values reported for SiO_2_ films grown on Si wafers [39].

**Table 2:** Results of the fitting analysis of the XPS O 1s and Si 2p emissions (x_c_ denotes the peak position of the O 1s contribution of silica).

	O 1s		Si 2p	area ratio
ALD cycles	x_c_ [eV]	FWHM [eV]	FWHM [eV]	O 1s/Si 2p

28	532.7	2.14	2.20	2.62
56	532.7	2.17	2.23	2.21
84	532.7	2.12	2.05	2.30
112	532.6	2.00	1.98	2.12

**Figure 3:**
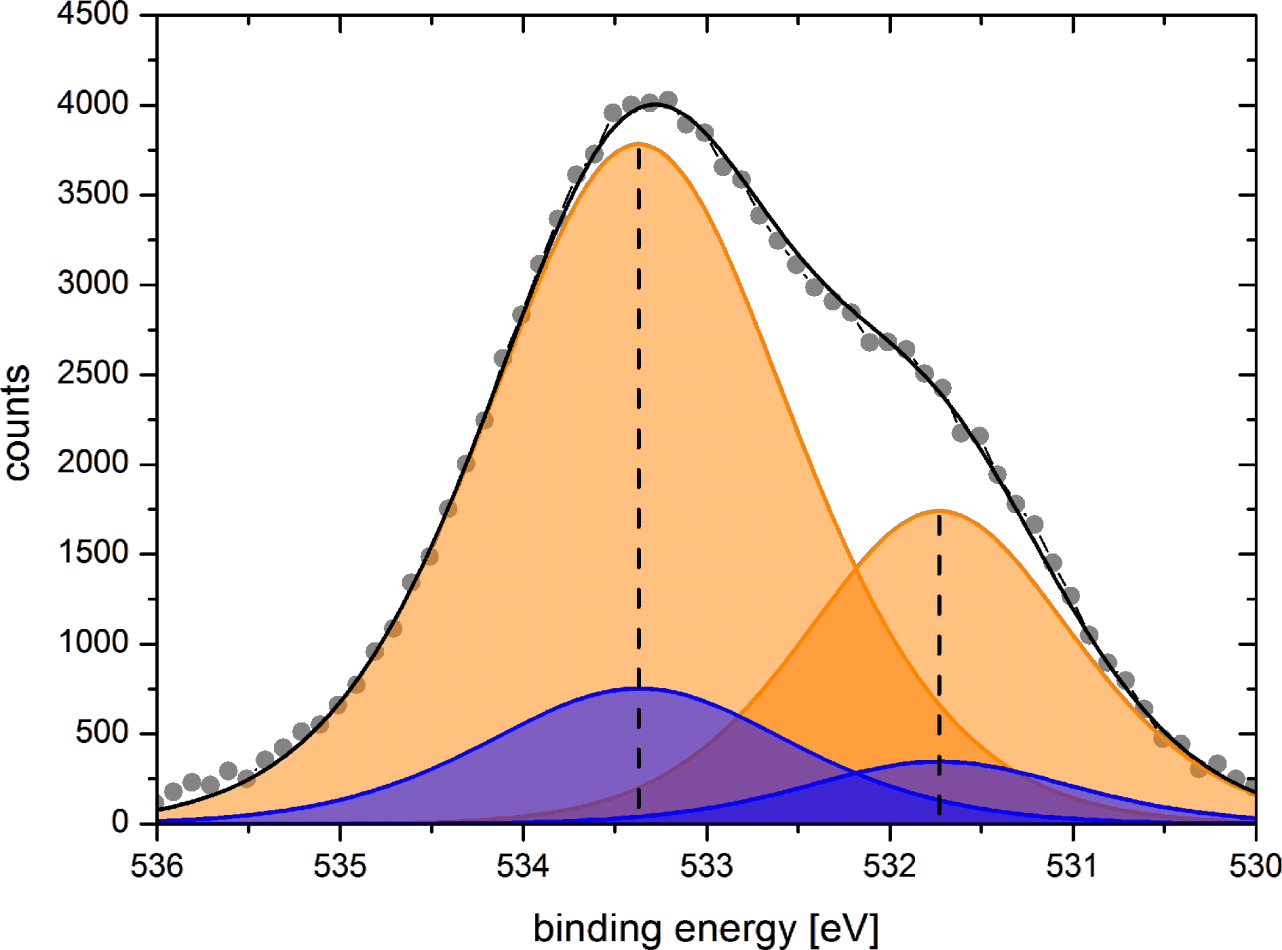
O 1s contributions of polycarbonate to the XP spectra of uncoated (orange, grey dots are measured counts) and SiO_2_-coated (blue, 28 cycles) membranes. For details see text.

While the XPS spectrum of the uncoated membrane is characteristic of polycarbonate [40], applying a small number of ALD cycles (e.g., 28) results in a superposition of polycarbonate and silica profiles. Figure 3 shows the O 1s contributions of polycarbonate to the spectrum of coated (blue) and uncoated (orange) membranes. The O 1s peaks of the uncoated membrane appear at 531.7 and 533.4 eV, and are attributed to double-bonded carbonyl oxygen [(O–(C=O)–O), 33%] and single-bonded oxygen [(O–(C=O)–O), 66%] in polycarbonate, respectively, in agreement with the literature [40]. After 28 ALD cycles the polycarbonate-related XPS peaks are strongly suppressed due to SiO_2_ film formation. After deconvolution of C 1s species for every sample, O 1s species with respect to the PC stoichiometric amounts, peak position and FWHM are fitted. The residual O 1s peak consits of SiO_2_, which fulfill the same peak position and FWHM criteria for all samples. This behavior strongly suggests that there is no interphase formation between the polycarbonate substrate and the deposited SiO_2_ films. The mechanism of SiO_2_ formation is proposed to be based on subsurface crystal growth resembling the previously observed growth behavior of alumina films on polymers [41].

### Morphology and conformity of ALD coatings

**Figure 4:**
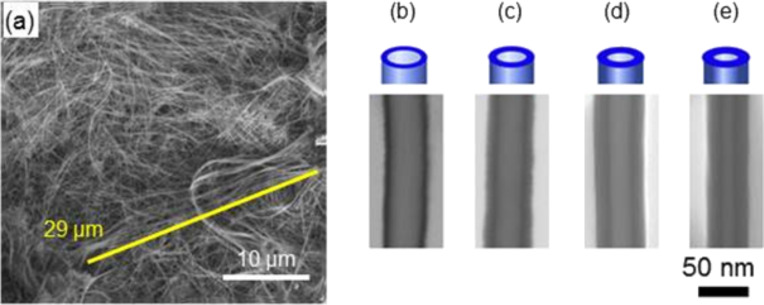
(a) SEM image of a bundle of highly-flexible SiO_2_ nanotubes after dissolution of the PC membrane. (b–e) STEM-in-SEM images of representative sections of SiO_2_ nanotubes after applying 28, 56, 84, and 112 ALD cycles, respectively.

The low magnification SEM image in Figure 4a shows a bundle of SiO_2_ nanotubes after 56 ALD cycles and subsequent dissolution of the polymer template. Most of the observed tubes are about 30 µm long, which corresponds to the initial thickness of the PC foil. This and the fact that the outer diameter is constant along the tubes evidence a conformal deposition process along the complete length of the nanochannels. Figure 4b–e display representative STEM-in-SEM images of samples exposed to 28, 56, 84, and 112 ALD cycles. These higher magnification images clearly demonstrate the tube-like shape of the fabricated nanostructures. Within each sample, all analyzed tubes exhibit constant wall thickness along their entire length, evidencing the excellent conformity of the SiO_2_ ALD process inside the nanochannels. From the STEM-in-SEM images, the outer and inner diameter as well as the wall thickness were determined for about 10–15 nanotubes per sample (Table 3).

**Table 3:** Characteristics of SiO_2_ nanotubes prepared by exposition to four different numbers of ALD cycles. Data from STEM-in-SEM images agree well with values deduced from SAXS measurements.

sample	ALD cycles	STEM-in-SEM (nm)			SAXS (nm)		
		outer diameter	inner diameter	wall thickness	outer diameter	inner diameter	wall thickness

Figure 4b	28	49 ± 5	31 ± 4	9.0 ± 6.2	50.0	39.0	5.3
Figure 4c	56	46 ± 3	23 ± 3	11.5 ± 4.1	48.6	28.5	10.0
Figure 4d	84	48 ± 3	16 ± 3	16.0 ± 4.2	49.3	20.1	14.6
Figure 4e	112	44 ± 4	11 ± 2	15.5 ± 4.3	50.4	14.8	17.8

**Figure 5:**
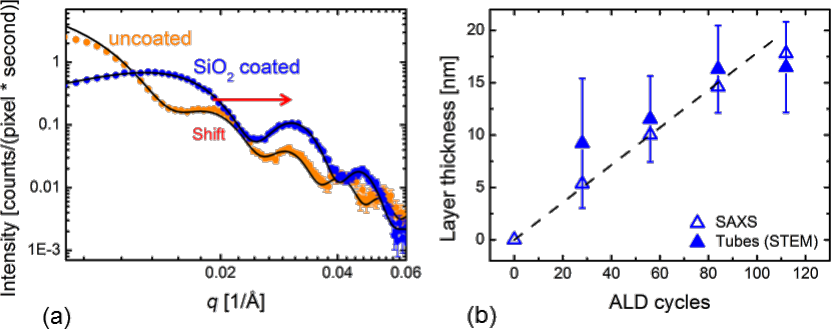
(a) Small angle X-ray scattering intensities as a function of the scattering vector *q* for an uncoated PC membrane (orange) and the same membrane coated with a 10 nm thick SiO_2_ layer after 56 ALD cycles (blue). (b) SiO_2_ layer thickness as a function of the number of ALD cycles according to analysis by SAXS (empty symbols) and STEM (solid symbols). The dashed line is a guide to the eye.

Complementary to SEM, the same samples were analysed by SAXS. Figure 5a shows the intensity profile deduced from the SAXS pattern for an uncoated reference membrane (orange) and for the membrane coated with SiO_2_ in 56 ALD cycles (blue). The distinct oscillations in both cases clearly indicate a narrow size distribution of the channels both before and after SiO_2_ coating. As expected, the oscillation maxima of the coated sample shift to larger *q*-values due to the reduced inner diameter of the pores. The preservation of the oscillations clearly show that the ALD process provides a homogeneous layer along the full length of all channels. A fit of the SAXS profiles with a cylindrical core–shell model for statistically distributed pores [35] allows us to deduce the inner and outer pore diameter and thus the thickness of the ALD-deposited SiO_2_ layer (Table 3). Additional parameters of the analysis are the polydispersity of the pores (6–8%), the layer roughness (8–10 Å) as well as the electron densities of the PC matrix (380 e/nm^3^), the SiO_2_ shell (560–610 e/nm^3^), and the empty core (0 e/nm^3^).

Figure 5b displays the values of the SiO_2_ layer thickness deduced from the SAXS analysis as a function of the number of ALD cycles (empty symbols). On the same graph, the wall thickness data of the nanotubes obtained by STEM-in-SEM analysis is displayed showing good agreement of both techniques. The linear increase of the thickness confirms the homogeneity and conformity of the layer and demonstrates the suitability of the ALD process to coat high aspect ratio nanochannels with SiO_2_ films of a few nanometres thickness in a tailored manner. This information is of great relevance for ALD applications on high-aspect-ratio nanochannels in general as well as for the development of further chemical modifications on the SiO_2_ coating.

### Wettability

**Figure 6:**
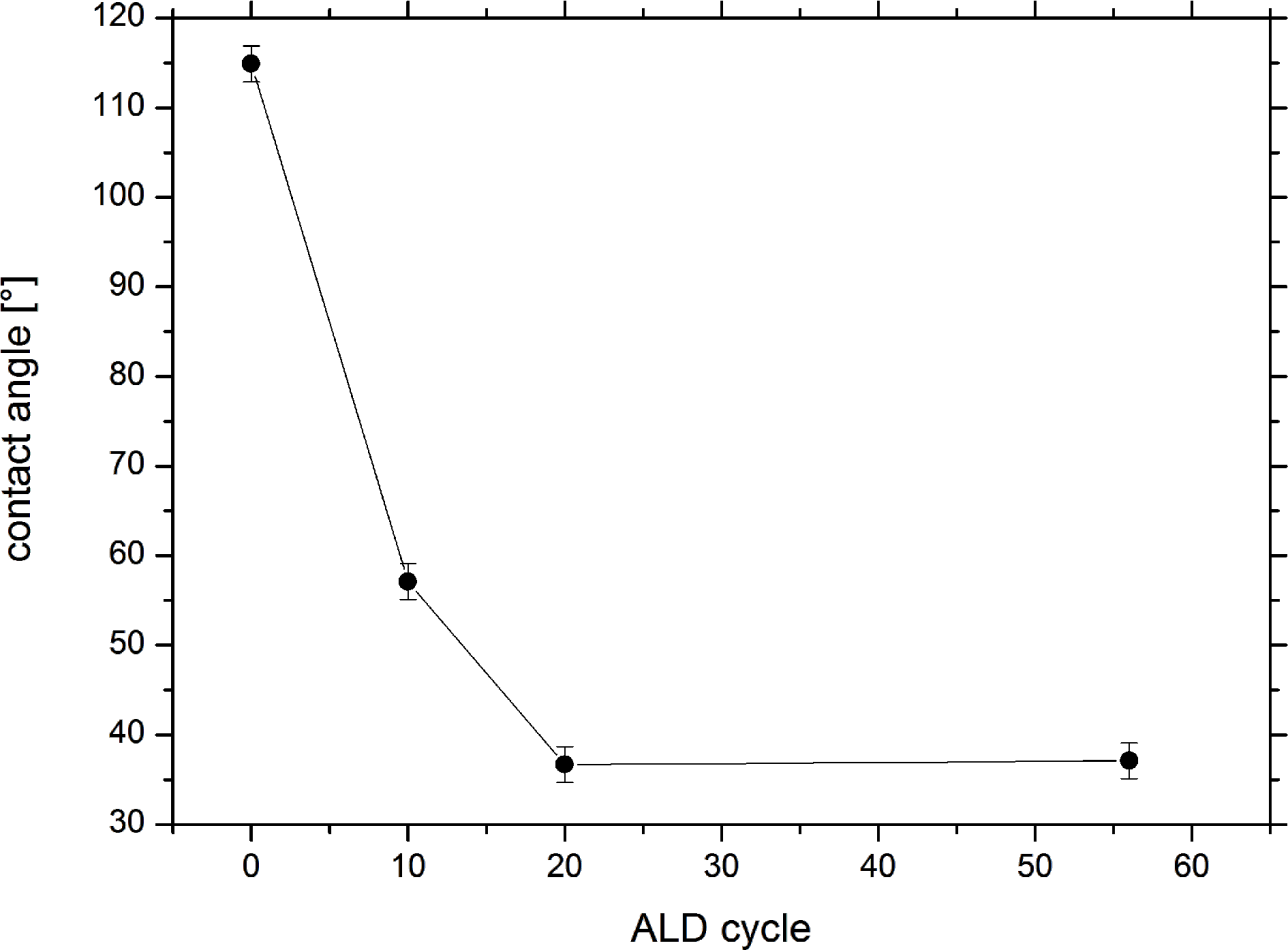
Contact angle measurements as a function of the number of ALD cycles. With increasing thickness of the SiO_2_ layer the samples become more hydrophilic.

The wettability of uncoated and SiO_2_-coated track-etched membranes was investigated by contact angle measurements. Figure 6 shows the contact angle as a function of the number of ALD cycles. The contact angle decreases with increasing thickness of the SiO_2_ layer, evidencing that the membrane surface changes its character from hydrophobic to hydrophilic. Above a layer thickness of about 7 nm, the surface shows excellent wettability.

## Conclusion

Cylindrical nanochannels in polycarbonate track-etched membranes with diameter of about 50 nm were conformally coated with SiO_2_ layers of thickness 5, 10, 15, and 18 nm by applying an ALD process at 325 K using SiCl_4_, water, and pyridine. The adjustment of the ALD process to almost ambient temperature guarantees the thermal stability of the PC membrane. FTIR and XPS analysis confirm the successful deposition of SiO_2_. HRSEM and SAXS investigations provide clear evidence of a conformal coating along the entire length of the nanochannels. The SiO_2_ coating significantly improves the wettability of the membranes. In conclusion, the ALD process proves to be most suitable for tailored coating of nanopores in polymeric track-etched membranes. By selecting a suitable number of ALD cycles, the process provides excellent control of size adjustment of nanopores. ALD is also flexible with respect to other surface modification and suitable for applications such as selective transport of ionic species or bio species through nanochannels and the development of novel nanopore sensors based on SiO_2_-surface modification.
